# Supplementary material for: Effects of M. tuberculosis and HIV-1 infection on in vitro blood-brain barrier function
Source: J Neuroinflammation. 2025 May 26;22:141. doi: 10.1186/s12974-025-03467-7 (PMC12107840; doi:10.1186/s12974-025-03467-7)
Supplement: Supplementary file 1 — Additional file 1: List of primers used in the study [file 12974_2025_3467_MOESM1_ESM.pdf]

| | | MMPs | | | Eotaxins | | | Interleukins | | | | | | | | | | MIPs | | VEGFs | | Fractalkine | IFNγ | TNFα | IP-10 | MCP-1 | RANTES |
|---|---|---|---|---|---|---|---|---|---|---|---|---|---|---|---|---|---|---|---|---|---|---|---|---|---|---|---|
| | | 2 | 3 | 9 | 1 | 2 | 3 | 1α | 1β | 2 | 4 | 6 | 8 | 10 | 12p70 | 13 | 17A | 1α | 1β | A | D | | | | | | |
| Astrocytes | Uninfected | 841.51 (433.07-1222.53) | 47.18 (23.72-63.22) | 0.27 (0-1.56) | 0.03 (0-0.28) | 0 | 0 | 0 | 0 | 0 | 0 | 51.31 (33.24-147.64) | 13.18 (5.74-63.05) | 0 | 0 | 0 | 0 | 0 | 0 | 822.64 (445.15-1258.98) | 0 | 2.09 (0-7.54) | 0 | 0 (0-0.38) | 0.45 (0-1.11) | 1023.53 (879.55-1519.51) | 0 (0-1.32) |
| | Mtb | 386.48 (190.17-764.48) | 46.47 (9.03-65.92) | 0 | 0 (0-0.16) | 0 | 0 | 0.14 (0-0.25) | 0 | 0 | 11.12 (0.35-15.07) | 333.71 (252.69-542.69) | 423.67 (106.77-629.9) | 0 | 0 | 0 | 0 | 0 | 0 | 545.84 (393.66-939.15) | 0 | 0 (0-4.18) | 0 | 0.03 (0-0.2) | 85.81 46.27-180.93) | 424.58 (368.53-503.66) | 2.21 (0-5.4) |
| | HIV-1 Bal | 412.33 (251.54-1160.12) | 15.73 (0-42.96) | 0 | 0 | 0 | 0 | 0 | 0 | 0 | 0 | 64.26 (0-128.74) | 2.09 (0-22.21) | 0 | 0 | 0 | 0 | 0 | 0 | 416.01 (337.46-1070.15) | 0 | 0 (0-6.99) | 0 | 0 (0-0.32) | 0 (0-0.32) | 797.33 (629.96-1033.57) | 0 (0-1.01) |
| | Bal + Mtb | 411.81 (251.54-1160.12) | 46.47 (0-64.7) | 0 | 0 | 0 | 0 | 0.01 (0-0.16) | 0 | 0 | 6.69 (5.21-12.84) | 380.36 (273.23-1795.42) | 439.78 (80.92-801.47) | 0 | 0 | 0 | 0 | 0 (0-2.18) | 0 | 502.91 (288.78-917.96) | 0 | 0 (0-3.38) | 0 | 0.06 (0-0.5) | 97.83 (28.66-174.37) | 411.16 (381.29-456.56) | 3.12 (0.28-9.66) |
| | HIV-1 VSV-G | 462.56 (182.46-764.48) | 44.48 (0-73.12) | 0 (0-0.1) | 0 | 0 | 0 | 0 | 0 | 0 | 0 | 167.89 (68.90-345.1) | 361.91 (66.2-819.31) | 0 | 0 | 0 | 0 | 0 | 0 | 428.6 (216.89-717.31) | 0 | 0 (0-2.49) | 0 | 0 (0-0.46) | 0.19 (0-4.12) | 530.59 (549.91-696.46) | 0.42 (0-1.32) |
| | VSV-G + Mtb | 244.39 (190.9-653) | 48.46 (22.5-88.21) | 0 (0-0.1) | 0 (0-0.04) | 0 | 0 (0-0.38) | 0.33 (0-0.6) | 0 | 0 | 17.14 (9.97-32.53) | 367.32 (276.99-1853.56) | 1187.24 (240.37-1531.24) | 0 | 0 | 0 | 0 | 0 (0-1.91) | 0 | 524.84 (391.66-826.38) | 0 | 0 | 0 | 0.38 (0.1-1.12) | 205.88 (90.08-222.72) | 420.23 (350.80-483.67) | 6.87 (3.35-16.98) |
| Endothelial cells | Uninfected | 381.45 (114.43-1005.87) | 17.58 (5.77-74.89) | 1.52 (0.1-2.69) | 1.57 (0.54-1.7) | 0 | 0.09 (0-0.21) | 2.48 (1.3-5.36) | 1.64 (0.95-3.92) | 0 (0-5.35) | 0 | 275.06 (79.27-353.88) | 7506.0 (2680.27-10043.42) | 0 | 0 (0-0.02) | 0 | 0 | 0 (0-0.68) | 0 | 1389.32 (742.92-3624.01) | 0 | 0 (0-13.3) | 2.48 (0-3.25) | 0.4 (0.24-2.11) | 4.93 (0.72-18.79) | 2020.75 (1236.46-3817.63) | 13.63 (11.71-66.87) |
| | Mtb | 136.05 (0-210.11) | 0 (0-26.25) | 0.1 (0-0.27) | 1.47 (0.38-1.74) | 0 | 0.38 (0-2.44) | 13.08 (7.77-32.17) | 167.86 (39.26-396.99) | 0 (0-8.95) | 7.25 (0-26.74) | 6263.56 (1150.61-44609.43) | 6404.59 (4860.79-12430.25) | 0 | 0 (0-0.35) | 0 | 0 (0-33.16) | 1.84 (0.85-2.56) | 3.84 (0-7.48) | 2101.29 (612.36-4763.36) | 0 (0-1.57) | 5.53 (0-15.1) | 1.98 (0-6.06) | 1.12 (0-3.06) | 65.26 (8.96-243.99) | 869.5 (647.14-1537.01) | 68.33 (16.58-202.83) |
| | HIV-1 Bal | 505.03 (243.26-1459.9) | 31.92 (3.3-110.44) | 1.52 (0-2.1) | 1.14 (0.62-1.46) | 0 | 0.21 (0-0.8) | 1.8 (0.96-4.78) | 1.07 (0-2.07) | 0 (0-3.85) | 0 (0-18.93) | 239.47 (130.94-322.69) | 3629.26 (1572.5-7861.8) | 0 | 0 (0-2.95) | 0 | 0 (0-1.64) | 0.6 (0-1.18) | 0 (0-2.69) | 1919.28 (440.48-3023.7) | 0 (0-1.66) | 0 (0-15.32) | 1.67 (0-5.33) | 0.54 (0.21-1.76) | 39.12 (1.07-58.41) | 1856.64 (1213.2-2606.88) | 14.11 (11.45-115.68) |
| | Bal + Mtb | 158.92 (61.3-216.8) | 0 (0-38.47) | 0 (0-0.1) | 0.93 (0.38-1.3) | 0 | 0.41 (0-1.76) | 11.52 (6.26-64.97) | 158.06 (35.22-464.25) | 0 (0-7.46) | 8.37 (0-34.45) | 85538.66 (1455.25-365609.44) | 8218.54 (5864.24-15724.75) | 0 | 0 | 0 | 0 (0-5.85) | 1.49 (0.88-2.79) | 4.39 (0-5.06) | 1935.71 (578.09-5590.25) | 0 | 6.53 (0-27.35) | 1.71 (0-3.89) | 0.94 (0.4-1.44) | 61.56 (16.97-1444.92) | 976.78 (663.59-1361.95) | 42.66 (25.01-408.63) |
| | HIV-1 VSV-G | 154.13 (33.45-1165.27) | 0 (0-76.83) | 0.27 (0-2.1) | 0.73 (0.51-1.64) | 0 (0-4.96) | 0.47 (0-0.79) | 2.82 (1.41-4.82) | 1.92 (0-3.29) | 0 (0-4.88) | 0 (0-22.66) | 439.0 (262.130-638.91) | 4305.38 (3734.7-8460.84) | 0 | 0 (0-1.18) | 0 | 0 (0-0.67) | 0.68 (0-1.08) | 0 (0-3.3) | 2649.64 (284.64-3010.82) | 0 | 0 (0-17.76) | 1.67 (0-3.91) | 0.63 (0.21-1.14) | 13.73 (0-93.07) | 1535.38 (815.43-3774.65) | 13.26 (8.21-143.24) |
| | VSV-G + Mtb | 92.2 (0-232.15) | 0 (0-36.45) | 0 | 1.46 (0.26-1.74) | 0 (0-5.85) | 1.01 (0-3.22) | 10.34 (8.89-62.92) | 126.38 (50.98-545.91) | 0 (0-5.8) | 0 (0-58.47) | 4994.78 (3988.97-135984) | 8269.78 (4824.17-17363.99) | 0 | 0 (0-0.58) | 0 | 0 (0-16.12) | 2.33 (1.51-2.79) | 3.53 (0-6.73) | 1749.21 (302.77-5149.17) | 0 (0-2.64) | 6.65 (0-30.41) | 1.98 (0-4.68) | 0.87 (0.36-4.27) | 30.24 (4.37-1709.64) | 756.89 (648.77-1397.81) | 44.56 (14.09-570.52) |
| Pericytes | Uninfected | 3178.22 (2824.99-3192.33) | 270.97 (230.14-355.07) | 5.94 (4.67-7.53) | 0.28 (0-0.55) | 0 | 0 | 0 (0-0.57) | 0 | 0 | 0 | 94.55 (18.89-256.67) | 1238.77 (832.6-2333.01) | 0 | 0 | 0 | 0 | 0 (0-0.94) | 0 | 1823.72 (767.84-2554.32) | 0 | 6.27 (1.35-15.59) | 0 | 0 (0-0.68) | 4.39 (0.05-6.5) | 551.06 (308.45-790.14) | 1.09 (0-4.63) |
| | Mtb | 2589.47 (2482.42-2661.7) | 205.42 (179.96-277.9) | 4.35 (1.52-5.05) | 0.24 (0-0.34) | 0 | 0.31 (0.18-0.48) | 0.24 (0.16-1.1) | 0 | 0 | 0.71 (0-23.87) | 982.51 (564.92-1977) | 2155.21 (1389.59-5072.29) | 0 | 0 | 0 | 0 | 13.22 (11.47-20.77) | 19.8 (12.79-20.8) | 1929.58 (300.8-3110.5) | 0 | 0 (0-5.88) | 0 | 0 (0-0.79) | 122.87 (43.29-175.15) | 421.53 (346.54-677.74) | 27.77 (19.27-56.19) |
| | HIV-1 Bal | 2799.88 (2686.63-3066.3) | 203.48 (191.69-251.11) | 4.89 (4.35-6.12) | 0 (0-0.3) | 0 | 0 | 0.12 (0-0.37) | 0 | 0 | 0 | 10.3 (9.26-186.04) | 1010.67 (751.25-1844) | 0 | 0 | 0 | 0 | 0 | 0 | 1494.14 (583.67-2154.04) | 0 | 7.05 (4.02-18.18) | 0 | 0 (0-0.4) | 0 (0-1.42) | 286.85 (252.94-446.02) | 0.49 (0-1.16) |
| | Bal + Mtb | 2641.79 (2308.58-2855.07) | 189.69 (162.79-218.72) | 3.66 (2.62-3.81) | 0.12 (0-0.55) | 0 | 0.48 (0.26-1.05) | 0.41 (0.36-1.52) | 0 | 0 | 9.94 (0-14.41) | 955.7 (815.01-2688.37) | 2386.06 (1305.25-6255.09) | 0 | 0 | 0 | 0 | 12.96 (12.83-23.42) | 21.34 (15.47-23.28) | 1706.93 (422.73-2026.59) | 0 | 1.35 (0-10.1) | 0 | 0.44 (0-0.94) | 109.35 (84.48-172.23) | 477.3 (324.29-664.76) | 37.72 (24.19-42.51) |
| | HIV-1 VSV-G | 2394.74 (2320.2-2626.19) | 219.1 (178.16-233.95) | 3.66 (3.15-5.58) | 0.16 (0-0.44) | 0 | 0 | 0.14 (0-0.66) | 0 | 0 | 0 | 8.21 (3.56-16.96) | 1996.42 (878.7-2367.42) | 0 | 0 | 0 | 0 | 0 | 0 | 1829.36 (429.24-1993.69) | 0 | 5.59 (0-13.86) | 0 | 0 (0-0.56) | 0 (0-0.32) | 414.37 (312.23-621.41) | 0 (0-1.11) |
| | VSV-G + Mtb | 2322.52 (1964.07-2610.31) | 152.29 (131.81-215.21) | 2.08 (0.27-4.89) | 0.26 (0.08-0.65) | 0 | 0.67 (0.53-0.9) | 0.81 (0.5-1.34) | 0 | 0 | 18.3 (5.92-25.99) | 2657.64 (859.91-4731.6) | 2601.74 (1486.96-6720.19) | 0 | 0 | 0 | 0 | 20.03 (14.24-34.46) | 24.36 (17.59-36.99) | 1442.07 (419.66-2171.2) | 0 | 3.23 (0-8.11) | 0 | 0.68 (0.32-1.12) | 207.7 (80.54-233.8) | 658.96 (445.67-831.6) | 40.56 (28.54-60.55) |
| Microglia | Uninfected | 65.67 (54.5-141.05) | 40.23 (29.33-47.18) | 3.94 (2.08-7.48) | 12.84 (2.37-19.35) | 0 | 0 | 0 | 0 | 0 | 0 | 362.76 (297.67-566.88) | 185.61 (175.95-211.78) | 0 | 0 | 0 | 0 | 0 (0-0.87) | 0 | 1179.83 (723.59-1741.01) | 0 | 0 (0-1.17) | 0 (0-0.17) | 0 (0-0.56) | 0.31 (0-1.53) | 631.61 (199.49-1169.77) | 1.28 (0-2.73) |
| | Mtb | 33.45 (16.6-81.28) | 103.86 (98.39-215.11) | 4.67 (2.19-5.94) | 5.42 (1.05-13.74) | 0 | 0.3 (0-1.16) | 0.17 (0-0.21) | 0 | 0 (0-3.28) | 0 | 1598.29 (1037.37-5038.85) | 1800.65 (835.44-1919.26) | 0 | 0 | 0 | 0 | 19.14 (13.2-45.01) | 12.86 (8.62-36.38) | 1236.95 (1097.84-2561.12) | 0 | 0.02 (0-3.04) | 0 (0-0.32) | 0 (0-1.76) | 0.9 (0.5-5.17) | 446.08 (146.82-873.37) | 12.25 (11.11-23.34) |
| | HIV-1 Bal | 67.09 (53.56-207.04) | 24.17 (19.28-27.28) | 2.69 (1.52-4.35) | 7.75 (3.43-14.13) | 0 | 0 | 0 | 0 | 0 (0-1.04) | 0 | 439.65 (346.92-698.17) | 209.12 (163.88-251.66) | 0 | 0 | 0 | 0 | 0.63 (0.37-1.71) | 0 | 1130.12 (692.8-1796.16) | 0 | 0 | 0 | 0 (0-0.94) | 0.42 (0-2.04) | 535.35 (258.47-1098.53) | 2.84 (0.27-4.53) |
| | Bal + Mtb | 26.63 (16.6-195.64) | 131.39 (92.74-225.02) | 2.66 (1.56-5.64) | 3.93 (0.76-15.37) | 0 | 0.47 (0-0.56) | 0 (0-0.42) | 0 | 0 | 0 | 1684.16 (1495.22-7203.22) | 1453.97 (999.81-2050.4) | 0 | 0 | 0 | 0 | 16.11 (10.49-44.21) | 6.28 (5.3-40.38) | 1631.8 (1145.16-2669.59) | 0 | 0 (0-1.35) | 0 (0-0.53) | 0.24 (0-1.41) | 1.11 (0.55-7.05) | 365.35 (125.2-859.02) | 10.56 (7.62-23.03) |
| | HIV-1 VSV-G | 61.3 (34.74-283.68) | 26.25 (15.83-38.6) | 2.69 (0.27-5.58) | 11.10 (0.05-26.4) | 0 | 0 | 0 | 0 | 0 | 0 | 735.55 (573.53-807.16) | 464.79 (213-673.57) | 0 | 0 | 0 | 0 | 0.51 (0.21-11.54) | 0 | 1446.92 (677.17-1612.87) | 0 | 0 | 0 | 0.1 (0-0.56) | 0.52 (0-3.23) | 533.15 (308.77-997.99) | 2.28 (1.47-8.71) |
| | VSV-G + Mtb | 207.68 (0-355.04) | 118.92 (81.23-154.19) | 3.66 (1.56-6.59) | 4.8 (2.9-29.31) | 0 | 0.85 (0-1.2) | 0.32 (0-0.53) | 0 | 0 (0-3.85) | 0 | 1648.12 (1573.15-6893.05) | 2722.99 (1282.57-3622.5) | 0 | 0 | 0 | 0 | 25.48 (18.9-50.68) | 10.72 (2.86-29.97) | 1055.14 (772.45-1558.59) | 0 | 0 (0-1.17) | 0 (0-0.18) | 0.32 (0-1.07) | 1.11 (0.32-13.08) | 445.36 (194.98-1264.34) | 21.72 (9.06-32.59) |
| BBB | Uninfected | 2210.94 (1785.2-3611.21) | 179.75 (125.15-217.48) | 9.16 (7.21-40.54) | 1.27 (0.95-1.53) | 0 | 0.07 (0-0.17) | 0.67 (0.12-1.34) | 0 (0-0.03) | 0 (0-4.97) | 0 (0-2.56) | 404.88 (164.59-889.3) | 1996.42 (964.75-3590.53) | 0 | 0 (0-0.01) | 0 | 0 | 0.3 (0-1.12) | 0 | 3239.51 (2807.15-5296.08) | 0 | 12.53 (9.3-18.34) | 0.59 (0-0.91) | 0.94 (0.71-1.18) | 3.77 (1.06-9.19) | 1399.33 (1062.53-1563.08) | 9.66 (2.22-13.97) |
| | Mtb | 1606.53 (1476.95-2789.18) | 186.72 (119.5-216.08) | 10.92 (6.8-43.67) | 1.37 (1.05-1.61) | 0 | 0.21 (0.16-0.34) | 2.39 (1.25-3.78) | 5.21 (3.69-12.96) | 0 (0-6.81) | 0 | 1921.78 (1034.63-3566.02) | 4137.56 (2562.99-5605.20) | 0 | 0 (0-0.65) | 0 | 0 | 1.86 (0.88-8.78) | 0 (0-6.63) | 4003.25 (3165.47-6152.16) | 0 | 20.07 (16.43-21.65) | 0.92 (0-2.03) | 1.28 (0.87-2.18) | 10.75 (2.85-25.73) | 1377.18 (883.04-1475.81) | 12.53 (4.5-17.76) |
| | HIV-1 Bal | 2211.38 (1670.04-2713.66) | 173.75 (119.02-226.72) | 6.97 (5.5-45.31) | 1.23 (1.05-1.58) | 0 | 0.11 (0.05-0.57) | 0.69 (0.34-1.15) | 0 (0-0.12) | 0 (0-4.11) | 0 | 317.67 (246.14-771.26) | 2280.69 (1682.98-3235.73) | 0 | 0 (0-1.07) | 0 | 0 | 0.69 (0.31-1.24) | 0 | 3561.02 (2850.91-5778.18) | 0 | 14.41 (7.72-18.24) | 0 (0-1.86) | 1.07 (0.5-1.9) | 2.51 (2.06-7.05) | 1313.79 (1022.85-1526.64) | 6.79 (2.91-7.84) |
| | Bal + Mtb | 1874.97 (1417.09-2611.99) | 185.72 (106.13-248.57) | 7.66 (4.83-48.53) | 0.95 (0.83-1.95) | 0 | 0.32 (0.14-0.44) | 2.72 (1.58-4.02) | 7.68 (5.27-15.87) | 0 (0-6.23) | 0 (0-6.68) | 1724.52 (563.03-3224.38) | 4476.68 (2262.59-6322.28) | 0 | 0 (0-0.75) | 0 | 0 | 2.02 (0.99-11.7) | 0.48 (0-8.94) | 4368.67 (3416.18-4431.74) | 0 | 26.26 (13.04-28.16) | 0.7 (0.16-1.91) | 2.18 (1.36-2.83) | 10.72 (4.32-40.27) | 1188.75 (744.71-1339.49) | 9.43 (4.14-16.04) |
| | HIV-1 VSV-G | 1735.48 (1224.89-2531.74) | 134.9 (81.17-214.04) | 7.66 (5.5-43.6) | 1.42 (0.96-1.61) | 0 | 0.24 (0.17-0.45) | 0.62 (0.33-1.08) | 0 (0-0.04) | 0 (0-3.76) | 0 | 374.61 (245.76-764.84) | 2243.92 (1798.02-2942.66) | 0 | 0 (0-0.95) | 0 | 0 | 1.22 (0.4-1.28) | 0 | 3588.97 (3164.57-5829.05) | 0 | 9.89 (5.51-18.9) | 0.04 (0-1.67) | 0.3 (0-1.25) | 4.48 (1.25-9.77) | 1089.83 (839.35-1492.59) | 7.02 (3.37-9.41) |
| | VSV-G + Mtb | 1785.65 (1353.44-2559.03) | 160.64 (94.16-225.13) | 6.11 (3.19-46.05) | 1.12 (0.79-1.61) | 0 (0-1.41) | 0.23 (0.19-0.33) | 3.09 (1.44-4.39) | 9.77 (4.81-16) | 3.52 (0-8.4) | 0 (0-8.7) | 2125.96 (552.02-4415.93) | 5322.68 (3513.98-10338) | 0 | 0 | 0 | 0 | 2.88 (1.57-9.74) | 0 (0-6.18) | 4601.21 (3443.04-5449.21) | 0 | 25.53 (12.25-26.22) | 0.76 (0.06-2.96) | 1.76 (1.03-1.91) | 14.07 (6.9-42.44) | 1341.3 (646.67-1795.38) | 8.92 (4.13-18.63) |
