# Supplementary figures and images for: Effects of M. tuberculosis and HIV-1 infection on in vitro blood-brain barrier function
Source: J Neuroinflammation. 2025 May 26;22:141. doi: 10.1186/s12974-025-03467-7 (PMC12107840; doi:10.1186/s12974-025-03467-7)

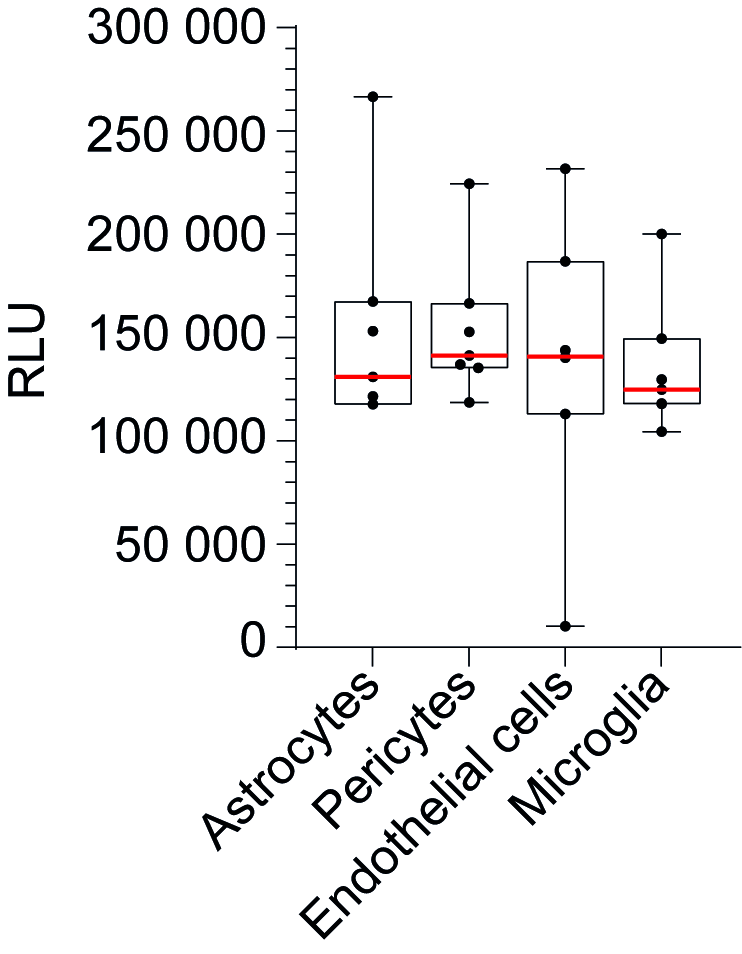

Supplement: Supplementary file 2 — Additional file 2: Flow cytometry gating strategy. Stained (eFluor™ fixable viability dye and FITC-conjugated anti-CD24 monoclonal antibody for cell viability and HIV-1, respectively), 4% PFA fixed and permeabilized cells were acquired on a LSRFortessa™ cell analyzer. A forward versus side scatter (FSC-A vs. SSC-A) gate (called “Cells”) is firstly used to identify the cell population and exclude debris. Onto the “Cells” gate, a forward scatter width (FSC-W) vs. forward scatter height (FSC-H) gate (called “Singlets”) is then used for multiplet exclusion. Onto the “Singlets” gate, a viability dye vs SSC-A gate (called “Alive”) is then used to discern between living and dead cells. Finally, onto the “Alive” gate, a HIV-1 (FITC) vs. Mycobacterium tuberculosis (RFP) quadrant gate is used to determine double negative (Mtb + HIV+), double positive (Mtb + HIV+) and single positive (Mtb + HIV- and Mtb-HIV+) cells. Compensation and fluorescence minus one (FMO) controls were used for every experiments [file 12974_2025_3467_MOESM2_ESM.tif]

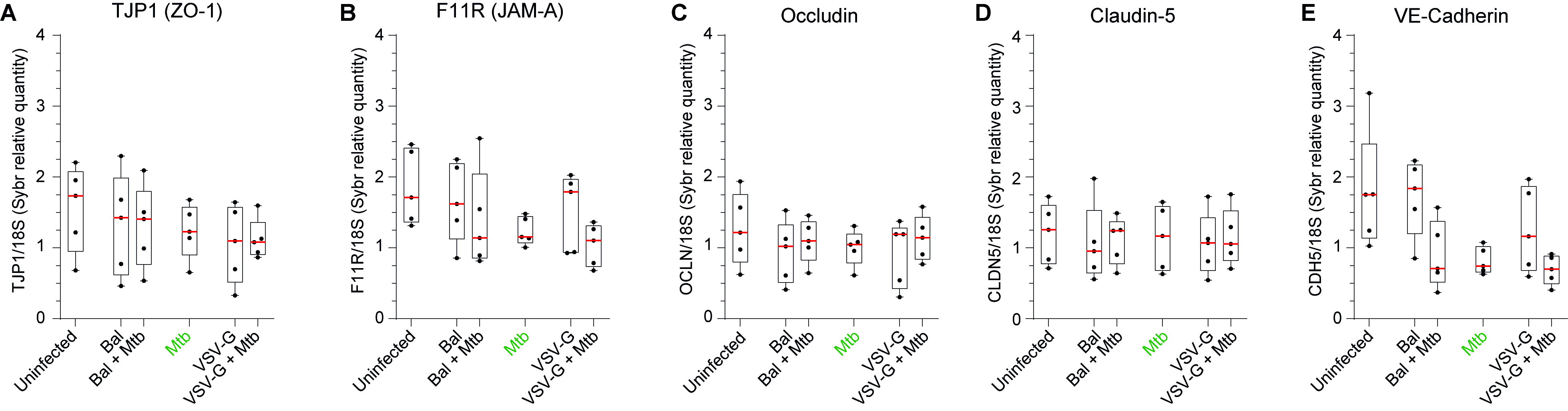

Supplement: Supplementary file 4 — Additional file 4: Effect of Mtb and HIV-1 infection on endothelial cell tight cell, and adherens junction gene expression. Endothelial cell gene expression of TJP1 (also known as ZO-1, panel A), F11R (encoding JAM-A, B), OCLN (Occludin, C), CLDN5 (Claudin-5, D) and CDH5 (encoding VE-Cadherin, E) was assessed by SYBR-Green Quantitative RT-PCR following infection with HIV-1 and/or Mtb. Results for 5 different experiments are presented as raw data normalized to the geometric mean of 18 S ribosomal gene expression [file 12974_2025_3467_MOESM4_ESM.tif]

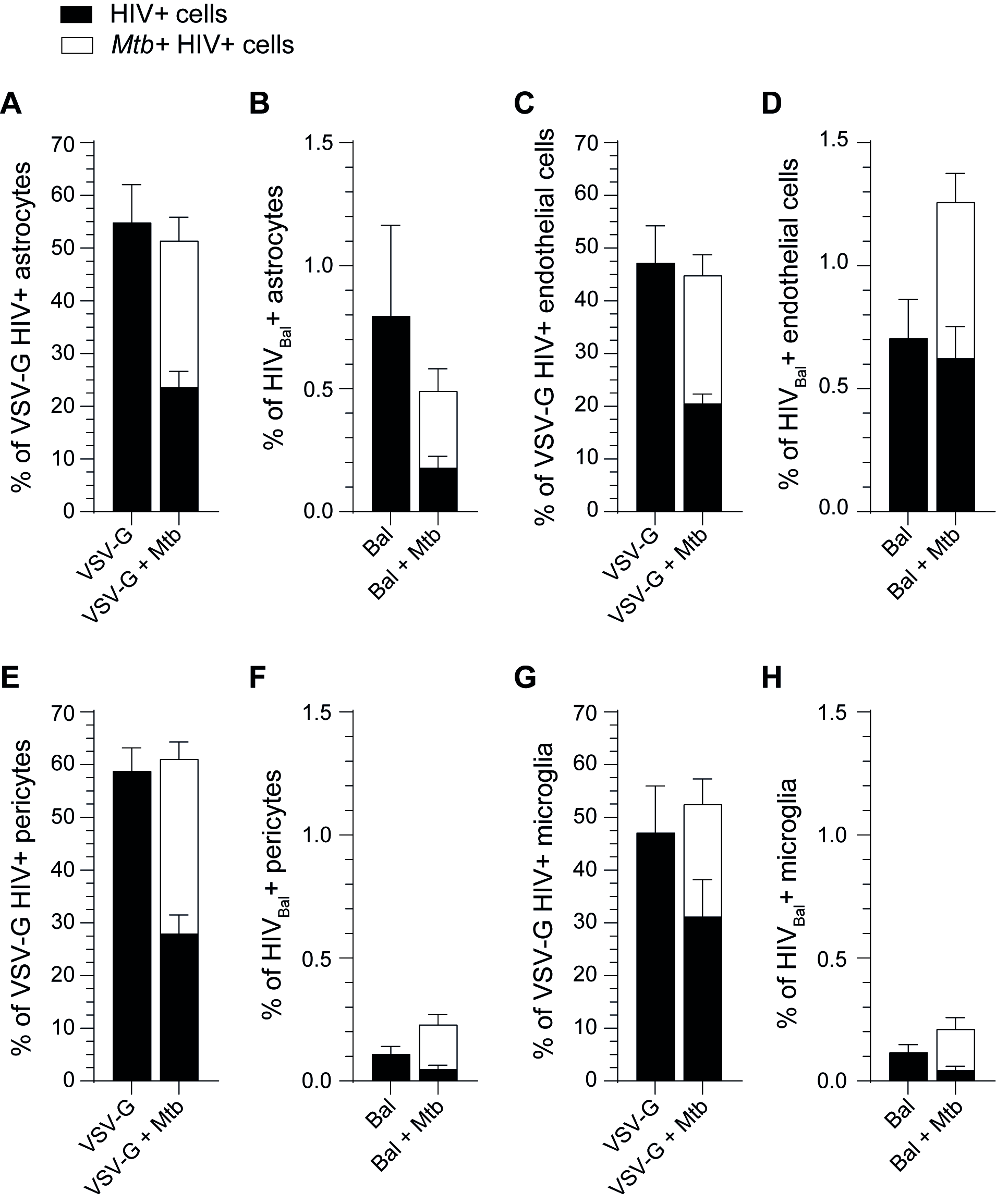

Supplement: Supplementary file 7 — Additional file 7: Soluble factors released from CNS cells and the BBB model upon Mtb and/or HIV-1 infection. The concentration of cytokines, chemokines, MMP and other soluble factors was measured after infection of CNS cells and BBB with Mtb and/or HIV-1 was determined by Luminex analysis as described in methods. The medians (IQR) of 7 (cells) or 5 (BBB) experiments are presented in raw data (i.e., pg/ml) [file 12974_2025_3467_MOESM7_ESM.tif]

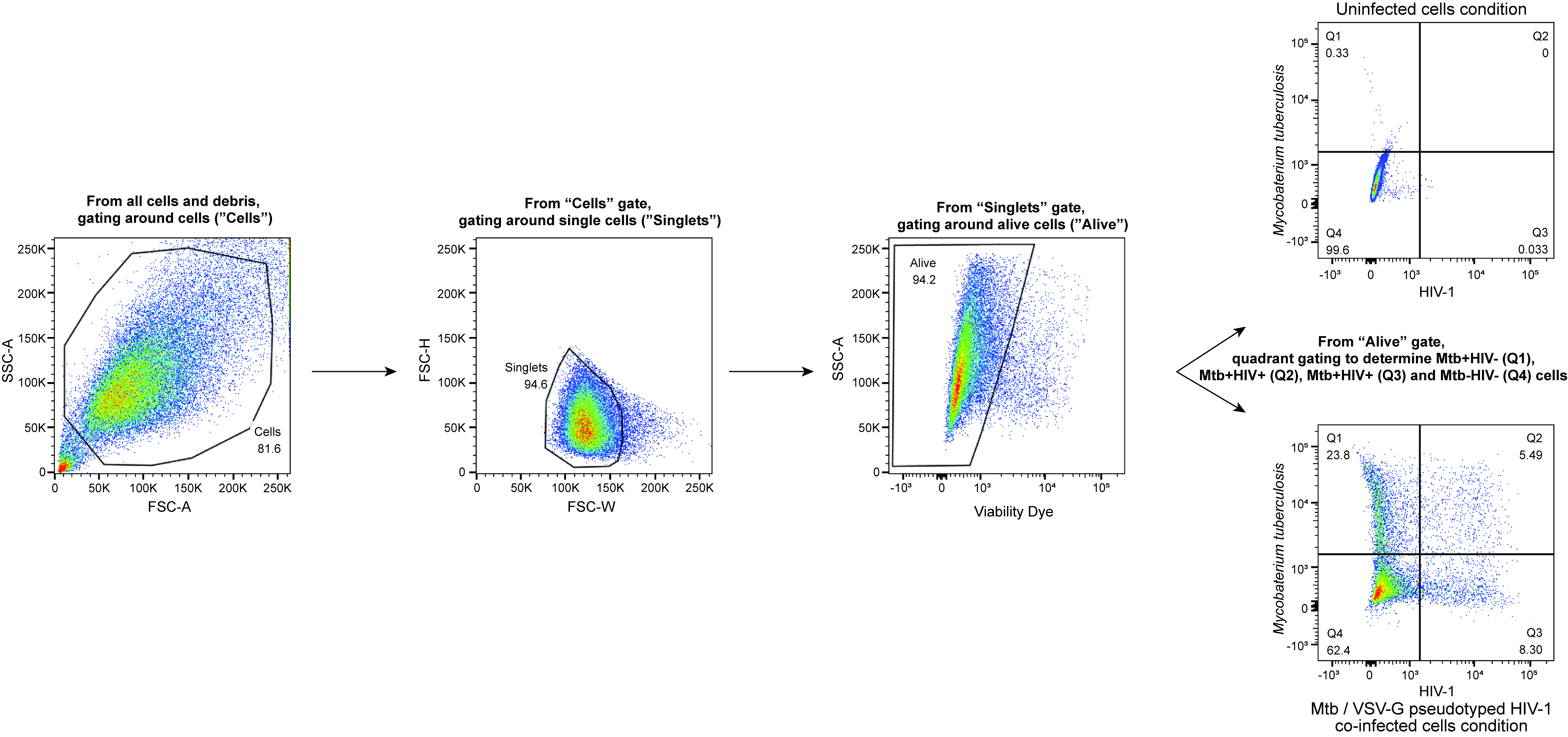

Supplement: Supplementary file 8 — Additional file 8: Summary of Mtb and/or HIV-1 effects on the BBB and CNS cells. “Mtb” column: effect of Mtb infection compared to the uninfected control. “HIV + Mtb” column: effect of HIV/Mtb co-infection compared to Mtb infection. + / -: increase / decrease. =: no effect or no increase. Bal / VSV-G: bystander effect / HIV-1 productive infection effect [file 12974_2025_3467_MOESM8_ESM.tif]
