# Supplementary material for: Effects of M. tuberculosis and HIV-1 infection on in vitro blood-brain barrier function
Source: J Neuroinflammation. 2025 May 26;22:141. doi: 10.1186/s12974-025-03467-7 (PMC12107840; doi:10.1186/s12974-025-03467-7)
Supplement: Supplementary file 3 — Additional file 3: HIV-1 infection of CNS cells. (A, B) Astrocytes, (C, D) hCMEC/d3 (endothelial cells), (E, F) HBVP (pericytes) and (G, H) HMC3 (microglia) were left uninfected, infected with (B, D, F, H) HIV-1Bal or (A, C, E, G) VSV-G pseudotyped HIV-1 for 24 h before being incubated for 3 h with Mtb followed by extensive washing. Cells were then incubated for 48 h before staining and analysis by flow cytometry. The percentage of HIV + and Mtb + HIV + cells are displayed in filled and opened bars, respectively. The mean and SEM for 5 to 6 different donors/passages are shown [file 12974_2025_3467_MOESM3_ESM.pdf]

		Astrocytes		Pericytes		Endothelial cells		Microglia		BBB	
		**Mtb**	**HIV+Mtb**	**Mtb**	**HIV+Mtb**	**Mtb**	**HIV+Mtb**	**Mtb**	**HIV+Mtb**	**Mtb**	**HIV+Mtb**
Mitochondrial activity imbalance		=	=	=	- (VSVG)	-	- (VSVG)	-	- (VSVG)		
ROS release		+	=	+	=	+	=	+	=		
Extracellular glutamate		+	=	+	+	=	=	+	=	+	=
MMP-	2	-	- (VSVG)	-	- (VSVG)	-	- (VSVG)	-	+ (VSVG)	-	=
	3	=	=	-	-	-	=	+	+	=	=
Interleukins	1β	=	=	=	=	+	=	=	=	+	+
	6	+	=	+	+ (VSVG)	+	+ (Bal)	+	=	+	+ (VSVG)
	8	+	+ (VSVG)	+	+	=	+	+	+ (VSVG)	+	+ (VSVG)
VEGF-A		-	-	+	=	+	=	+	=	+	+
IP-10		+	+	+	+ (VSVG)	+	=	=	=	+	+ (VSVG)
MPC-1		-	=	-	+ (VSVG)	-	- (VSVG)	-	-	=	=
IRE1	XBP1 splicing	+	+	+	+	+	+	+	+ (VSVG)		
	RIDD activity	+	+ (VSVG)	+	=	+	=	+	+ (VSVG)		
ATF6	BiP expression	-	-	-	=	+	+	-	- (VSVG)		
	uXBP1 expression	-	- (VSVG)	+	=	+	=	+	=		
PERK	CHOP expression	+	=	+	+	+	+	+	=		
Astrogliosis	C3 expression	+	+ (Bal)								
	S100A10 expression	=	+ (VSVG)								
Mtb entry	%		+		+		=		+		
	MFI		=		=		=		=		
Mtb growth	%		+ (VSVG)		=		=		=		
	MFI		=		+		+		=		
BBB permeability	Integrity									-	- (Bal)
	CFU										+
Cytotoxicity	Overall	+	+ (VSVG)	+	+	+	=	+	=		
	LT50		- (VSVG)		-		=		=		
Tight and adherens junctions	TJP1					-	=				
	F11R					-	-				
	OCLN					-	=				
	CLDN5					-	- (VSVG)				
	CDH5					-	-				
