# Supplementary material for: Effects of M. tuberculosis and HIV-1 infection on in vitro blood-brain barrier function
Source: J Neuroinflammation. 2025 May 26;22:141. doi: 10.1186/s12974-025-03467-7 (PMC12107840; doi:10.1186/s12974-025-03467-7)
Supplement: Supplementary file 6 — Additional file 6: Effect of HIV-1 and/or Mtb infection(s) on ER stress. sXBP1/uXBP1 (panels A-D), BLOC1S1 (E-H), CHOP (I-L), BiP (M-P), and uXBP1 (Q-T) transcripts were assessed by SYBR-Green Quantitative RT-PCR following infection(s) with Mtb, HIV-1 Bal and/or VSV-G pseudotyped HIV-1 of astrocytes (A, E, I, M, Q), hCMEC/d3 (B, F, J, N, R), HBVP (C, G, K, O, S), and HMC3 (D, H, L, P, T). Results for 5 to 6 different donors/passages are presented in raw data normalized to the geometric mean of 18 S ribosomal gene expression in box and whiskers plots. Asterisks denote statistically significant data determined by ANOVA with Dunnett’s correction for multiple comparisons (*P < 0.05, **P < 0.01, ***P < 0.001) [file 12974_2025_3467_MOESM6_ESM.pdf]

	Forward	Reverse
**Tight and adherens junctions**		
TJP1	5′-AGAAGGATGTTTATCGTCGCATT-3′	5′-CCAAGAGCCCAGTTTTCCAT-3′
F11R	5′-CAAGTCGAGAGGAAACTGTTG-3′	5′-TCTGACTTCAGGTTCAGAAGAG-3′
OCLN	5′-GTCCAATATTTTGTGGGACAAGG-3′	5′-GGCACGTCCTGTGTGCCT -3′
CLDN5	5′-GTGCTCTACCTGTTTTGCG-3′	5′-GACGGGTCGTAAAACTCG-3′
CDH5	5′-CAGCCCAAAGTGTGTGAGAA-3′	5′-CGGTCAAACTGCCCATACTT-3′
**Astrogliosis**		
C3	5′-AAAAGGGGCGCAACAAGTTC-3′	5′-GATGCCTTCCGGGTTCTCAA-3′
S100A10	5′-GGCTACTTAACAAAGGAGGACC-3′	5′-GAGGCCCGCAATTAGGGAAA-3′
**Endoplasmic reticulum stress**		
BLOS1	5′-CCCAATTTGCCAAGCAGACA-3′	5′-CATCCCCAATTTCCTTGAGTGC-3′
sXBP1	5′-GCTGAGTCCGCAGCAGGT-3′	5′-CTGGGTCCAAGTTGTCCAGAAT-3′
uXBP1	5′-CAGACTACGTGCACCTCTGC-3′	
BiP	5′-TCAGGCCAAGCCCAATACAG-3′	5′-TCCACGGTAGTGAGAGCCTT-3′
CHOP	5′-CAGAACCAGCAGAGGTCACA-3′	5′-AGCTGTGCCACTTTCCTTTC-3′
**Housekeeping gene**		
18S	5′-TAGAGGGACAAGTGGCGTTC-3′	5′-CGCTGAGCCAGTCAGTGT-3′
